# Antibacterial and Biofilm Production Inhibition Activity of *Thymus vulgaris* L. Essential Oil against *Salmonella* spp. Isolates from Reptiles

**DOI:** 10.3390/pathogens12060804

**Published:** 2023-06-05

**Authors:** Michela Galgano, Daniela Mrenoshki, Francesco Pellegrini, Loredana Capozzi, Marco Cordisco, Laura Del Sambro, Adriana Trotta, Michele Camero, Maria Tempesta, Domenico Buonavoglia, Piero Laricchiuta, Cristiana Catella, Annamaria Pratelli, Alessio Buonavoglia, Marialaura Corrente

**Affiliations:** 1 Department of Veterinary Medicine, University Aldo Moro of Bari, Sp Casamassima Km 3, 70010 Valenzano, BA, Italy; michela.galgano@uniba.it (M.G.); daniela.mrenoshki@uniba.it (D.M.); francesco.pellegrini@uniba.it (F.P.); marco.cordisco@uniba.it (M.C.); adriana.trotta@uniba.it (A.T.); michele.camero@uniba.it (M.C.); maria.tempesta@uniba.it (M.T.); domenico.buonavoglia@uniba.it (D.B.); cristiana.catella@uniba.it (C.C.); annamaria.pratelli@uniba.it (A.P.); 2 Istituto Zooprofilattico della Puglia e della Basilicata, Contrada San Pietro Piturno, 70017 Putignano, BA, Italy; lorepozzicadana@gmail.com (L.C.); laura.delsambro@hotmail.it (L.D.S.); 3 Zoosafari, Via dello Zoosafari, 72015 Fasano, BR, Italy; laris@libero.it; 4 Dental School, Department of Biomedical and Neuromotor Sciences, University of Bologna, Via Zamboni 33, 40126 Bologna, BO, Italy; alessio.buonavoglia85@gmail.com

**Keywords:** *Salmonella*, reptiles, *Thymus vulgaris*, antibiotic resistance

## Abstract

Salmonellosis is an infectious disease affecting both animals and humans. Antimicrobial resistant (AMR) and biofilm-producing *Salmonella* spp., frequently detected in reptiles (who can then act as asymptomatic carriers for warm-blooded animals), have developed resistance to biocides; this represents a warning for the emergence of biocide/antimicrobial cross-resistance. The aim of this study was to evaluate the efficacy of *Thymus vulgaris* L. essential oil (TEO) in inhibiting bacterial growth and biofilm production of *Salmonella* spp., which had been isolated from wild reptiles housed in a Zoo in Italy. The resistance profile against different classes of antibiotics showed that all the isolates were susceptible to the tested antibiotics, despite the presence of several AMR genes. All the isolates were also tested with aqueous solutions of TEO at different dilutions (5% to 0.039%). Interestingly, TEO proved effective both in inhibiting bacterial growth at low dilutions, with MIC and MBC values ranging between 0.078% and 0.312%, and in inhibiting biofilm production, with values ranging from 0.039% to 0.156%. TEO demonstrated effective bioactivity against the biofilm producer *Salmonella* spp., proving to be a valid disinfectant for the prevention of salmonellosis from reptiles, a possible source of infection for humans exposed to the reptiles’ environment.

## 1. Introduction

*Salmonella* spp. is considered one of the most important causes of bacterial gastroenteritis of public health significance worldwide, accounting for approximately 93.8 million cases every year [1] and can also rarely cause bacteriemia and systemic infections [2]. *Salmonella* spp. is a Gram-negative microorganism belonging to the *Enterobacteriaceae* family. The *Salmonella* genus consists of two species, *S. enterica* and *S. bongori*, and, to date, more than 2600 serovars belonging to *S. enterica* have been described worldwide as being ubiquitous and capable of causing illnesses in both humans and animals [3].

In the past, *Salmonella* spp. was considered almost exclusively a food-borne pathogen, but recently an increase in the number of human salmonellosis has been observed after direct or indirect contact with reptiles [4]. *Salmonella* spp., indeed, is a constituent of the intestinal microbiota of reptiles, which can host many serotypes simultaneously in the absence of symptoms, making these animals serve as reservoirs [5]. *Salmonella* spp. is often identified in captive reptiles and reports of salmonellosis related to pet reptiles are on the rise [6]. A high prevalence of *S. enterica* has been reported for pet reptiles, estimated to be 48–50% in lizards, 7–10% in chelonians, and 51–83% in snakes [7]. In Italy, a prevalence ranging from 46% to 57% has been reported [8]. Since the different prevalence may be linked to the intermittent excretion, every animal could be considered as a potential shedder [9]. Additionally, exotic pets, including lizards and snakes, have become increasingly popular, as has human interaction with animals during zoo visits or exhibition events, both of which could promote horizontal transmission of *Salmonella* spp. from reptiles to humans, and to other animals [10].

Most reptile-associated salmonellosis (RAS) appears to be responsible for serious disease and hospitalization, especially in children, elderly people, or immunocompromised persons [4]. The role of reptiles as reservoirs of antimicrobial resistant (AMR) *Salmonella* spp. has sparked increased interest in recent years [11]. The World Health Organization (WHO) considers AMR to be one of the most important health threats, and *Salmonella* spp. has been included in the priority list of twelve AMR bacteria [12]. A wide use of antibiotics against *Salmonella* spp. is described in the international pet reptile trade [13], and AMR has been reported in *Salmonella* spp. from captive reptiles, with important consequences for owners and breeders [14]. In addition to the increase in AMR, biocide resistance has also developed due to ability of *Salmonella* spp. to form biofilms, which may entail a worrying emergence of biocide/antibiotic cross-resistance [15]. Genes that code for AMR can sometimes be found on the mobile genetic elements (transposons) that facilitate transmission to other bacteria, thus increasing the risk of infection with AMR bacteria and posing a serious threat to public health worldwide [16]. Proper hygiene measures and effective disinfection are necessary to reduce the risk of *Salmonella* spp. infection [17]. However, the effectiveness of biocides depends on several factors, such as concentration, the state of the bacteria (i.e., the biofilms or planktonic cells), and the presence of interfering materials, such as organic matter. In recent years, the increase in cases of salmonellosis transmitted by reptiles (and the growing problem of AMR) impose us to find new molecules with antimicrobial activity. Among these, essential oils (EOs), aromatic oily liquid extracted from plants, have antimicrobial properties and have recently re-emerged as being natural and safe antimicrobials against pathogenic bacteria, including *Salmonella* spp., as well as against biofilm-embedded microorganisms [18,19,20]. The complex composition of EOs suggests that multiple mechanisms, probably acting synergistically, are involved in their biological effects [21]. These include the ability to alter the structure of the cytoplasmic membrane so as to have increased permeability, or to increase oxidative stress within microbial cells, leading to their death [22], in addition to having a potential inhibitory effect on intercellular communication systems (quorum sensing) or on the transcription of genes responsible for biofilm production [23]. Among these plant extracts, the Thyme Essential Oil (TEO) extracted from common thyme (*Thymus vulgaris* L.) exhibits high levels of antimicrobial activity (compared with other EOs, both in vitro and in vivo [20,24]) against *Salmonella* spp. [25].

In this context, the objectives of this study were to evaluate the AMR in *Salmonella* spp. isolates from wild reptiles housed in a Zoo, in addition to the effectiveness of TEO for the disinfection of terraria by inhibiting biofilm formation and bacterial growth.

## 2. Materials and Methods

### 2.1. Samples Collection

The sampling was carried out in February 2020 at the Zoological Park “Zoo Safari of Fasano”, Apulia (Italy), for a thesis project approved by the Ethical Commission of the Department of Veterinary Medicine (#approval number n. 9/20). The samples were collected from reptiles that were kept in the terrarium by the veterinary staff in charge of the Safari Zoo, in compliance with animal welfare and making use of the adequate containment techniques suitable for the various species.

Specifically, cloacal swabs were used to collect samples of feces from the reptiles and, where possible, feces were collected from the bottom of the cage. The exclusion criteria of the animals enrolled in the study were: (i) having a cloaca size that was too small for swabbing; (ii) clinical signs of disease or ecdysis (molt); (iii) any antibiotic treatment within the last 30 days. All the animals were declared healthy and suitable for sampling based on daily observations by the zookeepers, as well as a physical examination by the veterinarian responsible for the reptiles. A total of 13 swabs of the 50 reptiles in the reptile house were performed. Ten snakes were sampled: 4 royal pythons (*Phyton regius*); 2 royal snakes (*Lampropeltis getula*), one being a young subject identified as a “small royal snake”, and the other an adult subject identified as a “large royal snake”; 1 coral snake (*Micrurus fulvius microg albineus*); 1 bull snake (*Pituophis melanoleucus*); 1 false coral snake (*Lampropeltis triangulum*); and 1 pink boa (*Lichanura trivigata*). Among these, only the 3 royal pythons lived together in the same theca, while all the others came from individual theca. In addition, three saurians were sampled: 1 tiliqua (*Tiliqua occipitalis*), 1 gerrhosaurus major (*Gerrhosaurus major*), and 1 bearded dragon (*Pogona vitticeps*), all of which were kept in a single theca. The collected samples were stored at 4 °C and within 12 h they were sent to the bacteriology laboratory of the Department of Veterinary Medicine (DVM), University of Bari, Italy, and immediately processed. The characteristics of the sampled animals have been summarized in Table 1.

### 2.2. Bacteriological Analysis

All the samples were tested for *Salmonella* spp. Detection and identification, in accordance with Corrente [9]. The samples were pre-enriched in 9 mL of Buffered Peptone Water (BPW; Oxoid, Milan, Italy), and then incubated at 37 ± 1 °C for 18 ± 2 h. One mL of BPW from each sample was transferred into 9 mL of Selenite Cystine Broth (SCB; Oxoid, Milan, Italy) (incubated at 37 °C) and then into 9 mL of Rappaport–Vassiliadis broth (RVB; Oxoid, Milan, Italy), before being incubated at 41 ± 1 °C for 24 h. Cultures of RVB were inoculated on Xylose Lysine Deoxycholate agar (XLD; Liofilchem, Teramo, Italy) and incubated at 37 °C for 24 h. Colonies with the typical aspect of *Salmonella* spp. grown on XLD were inoculated on Triple Sugar Iron (TSI; Oxoid, Milan, Italy), and incubated at 37 °C for 24 h.

Suspected *Salmonella* spp. colonies (from 9 samples) were then processed, for biochemical identification (API 20E System^®^, bioMérieux, Rome, Italy).

### 2.3. PCR Characterization

The bacteria were tested with the *invA* gene-targeted PCR test to confirm their identification at genus level [26]. PCR was performed using DNA Thermal Cycler Gene Amp 9600 (Perkin Elmer Cetus, Norwalk, CT, USA). The samples were tested with the primers invA1: 5′-CGCGGCCCGATTTTCTCTCGGA-3′ and invA2: 5′-AATGCGGGGATCTGGGCGACAAG-3′, which amplify a 321 bp segment related to the *invA* gene [26]. 5 μL of template (DNA) of each sample was added to the reaction mixture as follows: PCR buffer (10×), 1.5 mM MgCl_2_, 200 μM of each triphosphate nucleotide, 0.1 mM of each primer, 2.5 U of Amplitaq Gold Polymerase (PerkinElmer Cetus, Norwalk, CT, USA), and distilled H_2_O to a final volume of 50 μL. The reaction mixtures were subjected to an initial denaturation cycle at 94 °C for 8 min, followed by 35 amplification cycles, each involving 1 min denaturation at 94 °C, followed by 1 min of annealing at 67 °C, 2 min of extension at 72 °C, and 1 final cycle of 8 min at 72 °C. For each PCR performed, 5 μL of sterile water was added as a negative control and a strain of *S. enterica* subsp. *Arizonae* from the collection of the DVM bacteriology laboratory as a positive control. The PCR products were visualized using an electrophoretic run on 1.5% agarose gel, with the aid of CHEMIDOC (Bio-rad, Milan, Italy). Identified bacterial isolates were stored at −80 °C for further serotyping and antimicrobial susceptibility testing.

### 2.4. In Vitro Antimicrobial Susceptibility

The disks of 23 antibiotics: Ampicillin (AMP; 10 μg), Ampicillin-Sulbactam (AMS; 20 μg + 10 μg), Piperacillin (PRL; 100 μg), Piperacillin-Tazobactam (TZP; 100 μg + 10 μg), Amoxicillin-Clavulanic acid (AMC; 30 μg), Ticarcillin-Clavulanic acid (TTC; 75 μg + 10 μg), Cefalexin (CL; 30 μg), Ceftazidime (CAZ; 30 μg), Ceftriaxone (CRO; 30 μg), Cefepime (FEP; 30 μg), Aztreonam (AZT; 30 μg), Trimethoprim-Sulfamethoxazole (SXT; 25 μg + 5 μg), Tetracycline (TE; 30 μg), Gentamicin (GN; 30 μg), Tobramycin (TOB; 10 μg), Nitrofurantoin (F; 5 μg), Enrofloxacin (ENR; 5 μg), Moxifloxacin (MOX; 5 μg), Ciprofloxacin (CIP; 5 μg), Nalidixic acid (NA; 30 μg), Chloramphenicol (C; 30 μg), Imipenem (IMI; 10 μg), and Meropenem (MRP; 10 μg), (Liofilchem, Teramo, Italy), were used to investigate the antimicrobial activity of all the *Salmonella* spp. Isolates in vitro, using the disk diffusion method (DDM). The antibiotics were selected based on available literature [27], and tested according to Clinical & Laboratory Standards Institute (CLSI) guidelines. The European Committee for Antimicrobial Susceptibility Testing (EUCAST) (http://www.eucast.org/clinical_breakpoints/, accessed on 2 January 2023) and the indications of CLSI (https://clsi.org/media/2663/m100ed29_sample.pdf, accessed on 2 January 2023) were used for the interpretation of the test after incubation at 37 °C for 24 h. Based on EUCAST interpretative criteria, the isolate strains were categorized as being either susceptible (S) or resistant (R).

### 2.5. Genomic Analysis

After biochemical identification, a single isolated colony from XLD agar was selected and inoculated into TSI, before being subjected to deeper investigation through whole-genome sequencing (WGS). DNA was extracted from all *Salmonella* spp. isolates using the DNeasy Blood and Tissue Kit (Qiagen, Hilden, Germany), in accordance with the manufacturer’s protocol. DNA concentration was estimated with a Qubit Fluorometer using Qubit dsDNA HS Assay (Thermo Fisher Scientific, Venice, Italy). For each isolate, a paired-end genomic library was prepared using a Nextera DNA Flex Library preparation kit (Illumina, San Diego, CA, USA). Sequencing was performed using the MiSeq Reagent Kitv2 (2250 bp) on an Illumina MiSeq platform (Illumina, San Diego, CA, USA). The paired-end raw reads were trimmed using Trimmomatic (GalaxyVersion0.36.6) [28], and then the draft genomes were assembled using SPAdes 3.12.0 [29]. The species identification on *S. enterica* was confirmed, which submitted the draft genome to the ribosomal Multilocus Sequence Typing (rMLST) database (https://pubmlst.org/rmlst/) [30]. Identification was performed at the serotype level. For that purpose, sequence typing from public databases for molecular typing and microbial genome diversity (PubMLST), according to *Salmonella* nomenclature in use at CDC, was considered [31]. Additionally, to identify AMR genes and plasmids, the draft genome of each strain was analyzed using the software ABRicate (Galaxy Version0.8), which includes different predownloaded databases [ARG-ANNOT [32], NCBIAMR Finder Plus [33], CARD [34], ResFinder [35], and Plasmid Finder [36].

### 2.6. EO—Compound Identification and Dilution Design

The choice of TEO and its concentration were based on data reported in the literature [20,37]. Commercially available 100% natural TEO (Specchiasol S.r.l., Bussolengo, VR, Italy), stored in a brown glass bottle at the temperature of 0–4 °C, was used. The percentage value of the components of TEO were detected using hyphenated gas chromatography with the mass spectrometry (GC/MS) technique [38,39], as reported by Galgano et al. [20] (Table S1). About twenty-five components were identified, comprising 98.7% of the total detected constituents. Thymol (47%), o-cymene (19.6%), and γ-terpinene (9%) were the main components, suggesting that the tested TEO was a thymol chemotype.

### 2.7. Evaluation of the Antimicrobial Activity of TEO

The antimicrobial activity of TEO was evaluated on logarithmic and stationary phases of *Salmonella* spp. isolates, preparing 10^8^ Colony Forming Units (CFU)/mL solution after 24 h of incubation for the logarithmic phase and standardized to 10^9^ CFU/mL solution for the stationary phase. Briefly, a bacterial suspension, containing approximately 5 × 10^8^ CFU/mL of each isolate, was prepared from a 24 h culture plate. For stationary phase, the strains were grown aerobically at 37 °C in BHI broth, with shaking at 150 rpm overnight, and the culture (early stationary growth phase) was used after standardization of the load for EO screens and drug exposure tests. The maximum level for each microorganism was 5 × 10^9^, 7 × 10^9^ [40]. Concentrations were estimated and standardized (~ 10^9^ CFU/mL), using the BPW (broth dilution assay) in agar dilution method [41].

#### 2.7.1. Broth Dilution Method and Minimum Inhibitory Concentration (MIC)

Determining the MIC with the microtiter broth dilution method of plant extracts was performed by means of serial dilution in 96-microtitrations well plates (Greiner bio-one, Frickenhausen, Germany) in accordance with the CLSI guidelines [42]. In each well, 100 μL of a cell suspension and 100 μL of TEO scalar dilutions of between 5% (*v*/*v*) up to 0.039% (*v*/*v*) in the final volume (or, expressed as *w/v*, from 54.1 µg/mL up to 0.42 µg/mL) were added. TEO dilutions in BHI broth were made in accordance with those laid out by Galgano et al., [20]. In addition, two samples were prepared as controls: a sample with BHI broth and DMSO (sterility control), and another sample with BHI broth with DMSO and bacterial inoculum, without EO. The plates were incubated at 37 °C for 24 h and 48 h, respectively, and the MIC values were then determined visually. The concentration that completely inhibited bacterial growth (the first clear well) was considered to be the MIC value.

#### 2.7.2. Minimum Bactericidal Concentration (MBC)

MBC, determined using the microtiter broth dilution method, was recorded as the lowest extract concentration capable of killing 99.9% of the bacterial inoculum after 24 h of incubation at 37 °C. The determination of MBC was performed using the method described by Ozturk and Ercisli [43]. Ten µL from the well of MIC value (as well as from wells above that value) were spread on XLD agar plates. The number of colonies was counted after 24 h and 48 h of incubation at 37 °C, respectively. The concentration of the sample that produced <10 CFU/mL was considered as the MBC value. Each experiment was repeated three times.

#### 2.7.3. Bactericidal and Bacteriostatic Effects

The averages that were generated from the MIC and MBC values at 24 h and 48 h, respectively, were used to estimate the bactericidal activity of TEO, expressed as the ratio of bactericidal concentration to inhibitory concentration (MBC/MIC). If the MBC/MIC ratio was low (1–2), the oil was considered bactericidal and was capable of killing 99.9% of bacteria; if the MBC/MIC ratio was high (4–6), it may not be possible to safely administer concentrations of EO that would kill 99.9% of bacteria, and it was thus considered bacteriostatic [44]. The test was performed from the logarithmic and stationary phases of bacteria that did not exhibit growth after treatment with TEO.

### 2.8. Disk Diffusion Method (DDM)

DDM was performed to evaluate the antibacterial activity of TEO at different concentrations against *Salmonella* spp., and results were then compared using the microtiter broth dilution method data, evaluated both in the logarithmic and stationary phase [45]. Bacterial cultures were spread over agar plates containing Mueller Hinton Agar (MH; Oxoid, Milan, Italy). Under aseptic conditions, empty sterilized discs (Whatman™ Antibiotic Assay Discs, 6 mm in diameter) were each impregnated with 10 μL of differing concentrations of TEO and placed on the agar surface. The inoculated plates were incubated at 37 °C for 24 h. Antimicrobial activity was evaluated by measuring the diameter of the growth inhibition zone surrounding the disc (mm) for eight tested concentrations (from 5% to 0.039%) against the isolated strains. The following intervals of inhibition were considered: (i) no inhibition; (ii) inhibition < 12 mm: weak activity; (iii) 12 mm ≥ inhibition < 20 mm: moderate activity; (iv) inhibition ≥ 20 mm: strong activity [46].

### 2.9. Detection of Biofilm and TEO Activity

According to Raad et al. [47], the overexpression of the genes responsible for biofilm formation starts at the end of the logarithmic phase. Biofilm production was evaluated with the Tissue Culture Plate method (TCP) as described by Christensen et al. [48], with some modifications. Organisms from logarithmic and from stationary phase cultures were tested in sterile 96-well flat-bottom polystyrene tissue culture-treated plates (Sigma Aldrich, Costar, St. Louis, MO, USA). Each well was filled with a two-fold dilution of TEO (starting from 5% to 0.039%, *v*/*v*) and then 100 μL of each bacterial suspension were added, up to a final volume of 200 μL in each well. Both a positive (100 μL of medium plus 100 μL inoculum) and a negative control (200 μL of medium) were used for each strain. After incubation at 37 °C for 24 h and 48 h, the solution from each well was ten-fold diluted in physiological solution and bacterial count from each dilution was performed in Plate Count Agar (PCA, Liofilchem, Teramo, Italy). All the plates were incubated at 37 °C for 24 h, and the inhibitory effect of TEO on biofilm production was evaluated. The empty wells were washed four times with 200 μL of phosphate buffer saline ((PBS) pH 7.2) to remove free floating bacteria. The bacterial biofilm adhering to the wells was fixed with 2% sodium acetate and stained with crystal violet (1%). The excess stain was removed using deionized water, and the plates were then dried in an inverted position to evaluate the presence of a visible film at the bottom of the wells.

To evaluate the growth and the biofilm production, all *Salmonella* spp. isolates were inoculated in BHI with TEO from 5% to 0.039% (*v*/*v*), and without TEO in the same plate, and incubated at 37 °C for 24 h.

### 2.10. Data Analysis

Statistical analysis was performed using the software R version 4.2.2 (R Foundation for Statistical Computing, Vienna, Austria). A *p*-value < 0.05 was considered as statistically significant. The CFU measurements for each dilution of TEO were analyzed as continuous quantitative variables, and the normality distribution was evaluated using the Shapiro–Wilk normality test. Data were analyzed with a One-way Analysis of Variance (ANOVA), followed by a Bonferroni test as a post hoc test (statistical significance set at 0.05).

## 3. Results

### 3.1. Bacteriological Analysis

Eight out of the ten tested snakes, and one out of the three tested saurians, were positive for *Salmonella* spp., which was confirmed using the Analytical Profile Index test (API 20E System^®^, bioMérieux; Rome, Italy). The respective genomic analysis confirmed the identification of different subspecies: five *S. diarizonae*; one *S*. *salamae*, one *S. enterica* and one *S. houtenae*. The characterization of isolates and the results of serotyping using MLST have been reported in Table 2.

### 3.2. AMR

The detected *Salmonella* were susceptible to all the tested antibiotics (Table S2). However, they possessed at least one of several genes encoding multidrug efflux pumps: *acrB*; *armB*; *CRP*; *mdtK*; *baeR*; *msbA*, which is responsible for exporting multiple antibiotics and toxic compound from the inside to the outside of bacterial cells; *MarA*, a gene of multiple antibiotic resistance; *AAC(6’)-Iy*, which is an aminoglycoside resistance gene; and *bacA*, which confers resistance to bacitracin. Regarding the two identified genes that code for fosfomycin resistance, *E.coliUhpT* gene was found in all strains, while *E.coliGlpT* was absent in *Salmonella* 6 and 8. The *SoxR* and *SoxS* genes, associated with multidrug resistance and prevention of oxidative damage, were detected in all the tested isolates, except for *Salmonella* 8. In contrast, the *sdiA* gene, associated with multidrug resistance and increased levels of *acrAB* was detected in six out of the nine strains (Table 2).

### 3.3. TEO Antibacterial Activity

The bactericidal and/or bacteriostatic properties of TEO was evaluated using MIC and MBC at two different times and in both phases, logarithmic and stationary (Table 3). In the logarithmic phase, the MIC and MBC values coincided at 24 h, ranging from 0.078 to 0.312 µL/mL. Most of the isolates showed a MIC and MBC values of 0.156 µL/mL, except for *Salmonella* 1 and *Salmonella* 9, which showed MIC and MBC values of 0.312 and 0.078 µL/mL, respectively. At 48 h, however, a decrease in MIC value was observed for strains 1, 4, 5, 6, 8, and 9, while the MBC values were constant.

In the stationary phase, the values were very similar to those of the logarithmic phase. After 24 h, MIC and MBC values coincided in the logarithmic phase, except for *Salmonella* 7 (MIC and MBC values 0.312 µL/mL). At 48 h, however, an increase in MIC values was observed only for *Salmonella* 1, while MBC remained constant.

The DDM test showed that, in both the logarithmic and stationary phase, only the 5% concentration proved effective, having inhibition values ranging from ≥12 mm to <20 mm (Table 4), which indicated a moderate action of TEO. In the logarithmic phase, the mean values of the detected inhibition alone for *Salmonellae* 1, 3, 7, 8, and 9 ranged from 12 mm to 15 mm; in the stationary phase, by contrast, the mean values of the detected inhibition alone for *Salmonellae* 1, 3, 6, 7, 8, and 9 ranged from 12 mm to 16.75 mm.

### 3.4. Biofilm Production and TEO Activity

**Figure 1:**
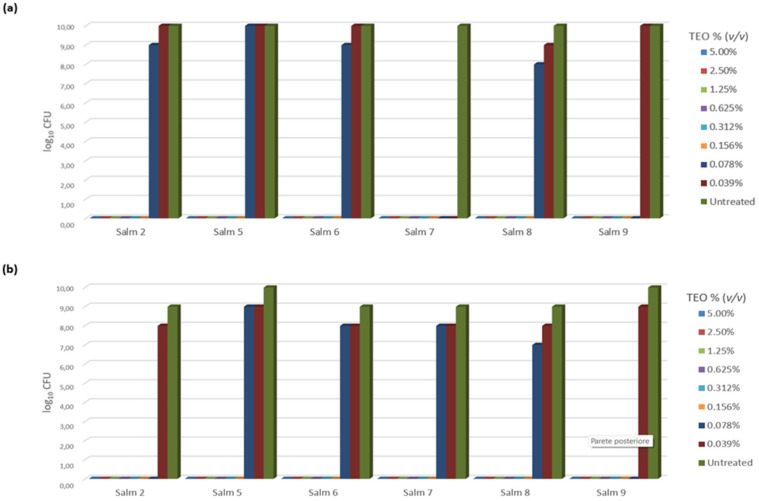
Efficacy of different concentrations of TEO (*v*/*v*) on inhibiting biofilm production *Salmonella* isolates, evaluated after 48 h incubation: (**a**) logarithmic phase at 48 h of incubation; (**b**) stationary phase at 48 h of incubation.

Six isolates were identified as biofilm-producing bacteria. The ability of different TEO concentrations (*v*/*v*) to inhibit biofilm production was reported in Figure 1. The inhibition of the biofilm production of *Salmonella* strains was expressed as the absence of biofilm after violet crystal staining; where biofilm production was not inhibited, the bacterial growth (expressed as log_10_ CFU/mL) was evident.

The normality of distribution was evaluated using a Shapiro–Wilk normality test (W = 0.97958, *p*-value < 0.05). TEO totally inhibited bacterial growth at 5.00%, 2.50%, 1.25%, 0.625%, 0.312%, and 0.156% concentration (*v*/*v*) of all *Salmonella* isolates tested during logarithmic phase, once incubated for 24 and 48 h (*p* < 0.05) (Figure 1a). However, lower concentrations did not perform so well. Specifically, a TEO concentration of 0.078% (*v*/*v*) completely inhibited biofilm production in *Salmonellae* 7 and 9 (*p* < 0.05), while a 10^1^ CFU/mL reduction in microbial growth (compared to the positive control) was observed for *Salmonellae* 6, and 10^2^ CFU/mL (*p* < 0.05) for *Salmonella* 8. No inhibition at all was detected for *Salmonella* 5 at 0.078% (*v*/*v*). The lowest TEO concentration, 0.039% (*v*/*v*), completely inhibited biofilm production of *Salmonellae* 4 and 7 (*p* < 0.05), while also significantly reducing *Salmonella* 8 of 10^1^ CFU/mL (*p* < 0.05). No inhibition at all was detected for *Salmonellae* 2, 5, 6, or 9.

In the stationary phase, similar results were observed; this was except for *Salmonella* 2, in which no bacterial growth was observed at a 0.078% concentration (*v*/*v*), as well as for *Salmonella* 7, which grew at 0.039%, but at 0.078% concentration (*v*/*v*) still showed a significant reduction (*p* < 0.05) (Figure 1b).

## 4. Discussion

Reptiles and amphibians are estimated to be responsible for 11.7% and 7% of all *Salmonella* spp. infections, respectively, in the United States and Europe [49,50]. Sauteur et al. [51] examined studies published from 1965 to 2012 that described RAS in children younger than 18 years of age, with a total of 182 cases identified. In that study, all the tested reptiles were clinically healthy, confirming the asymptomatic carrier status of the animals. All *Salmonella* spp. isolates belonged to *S. enterica* and corresponded to different subspecies. The exact serotyping of *Salmonella* spp. isolates from reptiles may not always be correct, due to both the presence of a wide variety of serotypes and because these serotypes may not coincide with the main epidemiologically known serotypes of humans and pets. In our study, only *Salmonella* 9 was not identified, while all the other isolated strains had already been reported in the literature, confirming that cold-blooded animals are the main reservoir for the *houtenae*, *diarizonae*, *salamae*, and *arizonae* subspecies [52]. However, the most common serotypes in humans and farm animals belong to *S. enterica* subsp. *Enterica*, which is to say *S. enteritidis*, *S. typhimurium*, *S. choleraesuis*, *S. infantis*, *S. derby*, *S. dublin*, *S. hadar*, and *S. virchow* [53]. Although these serotypes were not included among those identified by PubMLST, evidence of their hazard to humans has been supported by Wybo et al. [54], who reported a case of reptile-associated meningitis in a 2.5-month-old infant, caused by *S. enterica* subsp. *houtenae* serotype *44:z4*, *z23:-*. In addition, the *Salmonella* spp. detected in this study have previously been detected in reptiles as a potential source of transmission of multidrug-resistant *Salmonella* isolates [14]. Furthermore, although both *S. enterica* subsp. *salamae* and *S. enterica* subsp. *hountenae* have a poor ability to invade host cells, they can cause infections in immunocompromised individuals [52]. By contrast, *S. enterica* subspecies *diarizonae* has been increasingly associated with infections in humans, especially after direct contact with reptiles [55]. Furthermore, reptiles have also been shown to harbor MDR *Salmonella* spp. [56,57], and the increasing prevalence (due to the extensive use of antibiotics in reptile breeding and during long-distance transport [14]) is a potential cause for public health and safety concerns, due to the increased severity of the disease, the duration of hospitalizations, and the higher health costs [4]. Interestingly, in this study, and in contrast from what has been reported in the literature, the identified *Salmonellae* spp. showed no phenotypic resistance to antibiotics during common screening with DDM [9,14]. Furthermore, our findings disagreed with the presence of the resistance genes detected within the genome of the tested strains. Indeed, WGS results showed that the isolates possessed a high number of AMR genes. For each selected drug category (β-lactam, macrolide, carbapenem, aminoglycoside, quinolone, rifampicin, and tetracycline), the corresponding resistance genes were detected. In addition, resistance genes to fosfomycin, aminocoumarin, nitroimidazole, and phenicol, as well as genes responsible for resistance to multidrug and disinfectant agents, such as those encoding for the multiple efflux pumps, were detected. Regarding the resistance genes identified, it is important to note that for those genes encoding multidrug efflux pumps, a single multidrug efflux pump can eject multiple antibiotics contributing to MDR [58], while the “multiple antibiotic resistance” (*MarA*) gene participates in the control of several genes involved in resistance to not only antibiotics, but also oxidative stress, organic solvents, and heavy metals [59,60]. The emergence of cross-resistance is well documented in the literature, and has often been related to the overuse of these molecules, in addition to the potential selection pressure they may confer [58,61]. It is important to underline that since *Salmonella* spp. is excreted in feces, it could not only contaminate reptile skin, but also the environment and surfaces during exhibits, and this contamination can be a source of infection to humans, including both those who handle reptiles and those who are exposed to the reptile’s environment [8,62].

In this context, valid alternatives for the disinfection of the environments in which these animals are kept must be sought. Phytochemical constituents of EOs hinder the microbial resistance process and are distinguished by an exceptional combination that can inhibit biofilm development processes, including both phenols [63] and sulfur-containing compounds [64]. The antimicrobial activity of EOs, although influenced by their composition, was largely demonstrated, being shown to act on the lipid bilayer of the cell membrane, negatively affecting the cell cycle of bacteria, and inhibiting protein synthesis [20]. Molecular docking simulations have also demonstrated that TEO was able to inhibit topoisomerase II (as well as both the DNA and RNA polymerase) activity of bacteria, compromising the DNA replication and transcription processes [65].

Regarding the ability of TEO to inhibit both the growth and biofilm production of *Salmonella* spp. in the logarithmic and stationary phases, this study sought to evaluate and identify the most effective concentration for environmental disinfection. Our results agreed with data in the literature [25,66], and showed a remarkable efficacy against several subspecies of *S. enterica*. Even at low concentrations, TEO demonstrated antibacterial efficacy against *Salmonella* spp. strains carrying resistance genes, with MIC and MBC values between 0.078% (0.84 µg/mL) and 0.312% (3.37 µg/mL). In both phases, the MBC/MIC ratio was low, i.e., 1–2, allowing us to conclude that the action of TEO is bactericidal [43]. Furthermore, TEO inhibited biofilm production, with a concentration ranging from 0.039% (0.42 µg/mL) to 0.156% (1.68 µg/mL); this confirmed that TEO, as had been previously reported [23], was an excellent adjuvant for the disinfection of the environments. Since biofilm-producing bacteria are more resistant to antimicrobial agents, the use of those molecules that are able to inhibit biofilm production is a valid system for sanitizing environmental surfaces.

DDM provided discordant data with those reported by Ed-Dra et al. [25], and at the maximum TEO concentration, we observed only intermediate inhibition diameters. However, compared to the dilution methods, DDM is less reliable due to the difficulty of the oil to spread from the disk into the agar, invalidating the efficacy of the test.

The limits of our study were the small sample size, which made the study unsuitable for statistical investigation, and a discrepancy resulted from the two different testing methods used. The disk diffusion test, agar dilution test, well diffusion test, and broth microdilution are currently employed [67], but no standardized methods to screen the antibacterial activities of EOs are available. Our data, compared with the literature, suggest that the broth microdilution method may be more sensitive than DDM for detecting the antimicrobial action of Eos, in addition to producing more accurate and reproducible results in different trials [67].

## 5. Conclusions

In conclusion, effective sanitary measures to counteract RAS must be employed, but considering the high prevalence of healthy carriers and MDR strains in reptiles, antibiotic prophylaxis is neither useful nor appropriate to tackle this problem [4]. Our study adds an important contribution to the literature, providing useful data on the bioactivity of TEO in the control of AMR and biofilm-producing *Salmonella* spp. Our results showed that TEO could be a valid disinfectant for the disinfection of theca and terrarium, being able to inhibit *Salmonella* spp. growth, both in stationary and in logarithmic phases, and thus represents a useful approach for the prevention of salmonellosis from reptiles, improving human and animal health and welfare while safeguarding the environment.

## Figures and Tables

**Table 1:** Tested animals, their characteristics and sampling place.

Reptiles	Animals	Sex	Weight (g)	Theca	Sample
**Snakes**	Royal python (P1)	F	1500	Shared *	CS
	Royal python (P2)	M	850	Shared *	CS
	Royal python (P3)	F	1880	Shared *	CS
	Royal python (P4)	F	400	Single	CS
	Small royal snake	M	310	Single	CS
	Large royal snake	F	1290	Single	CS
	Albino coral snake	M	156	Single	CS
	Bull snake	M	110	Single	CS
	False coral snake	F	400	Single	CS
	Pink boa	F	200	Single	CS
**Saurians**	Tiliqua	F	386	Single	CS + Feces
	Gerrhosaurus major	M	360	Single	CS
	Bearded dragon	F	320	Single	CS + Feces

CS: cloacal swab; F: female; M: male; * Royal pythons P1, P2, P3 shared the same theca.

**Table 2:** Identification, typing and characterization of detected *Salmonella* spp. in reptiles.

Positive Animals	Detected *Salmonella*			
	ID Sample	Species and Subspecies	Serotype	Antimicrobial Resistance Gene ^1^
**Large royal snake**	*Salmonella* 1	*S. enterica* subsp. *diarizonae*	51:k:z35	*marA*; *soxS*; *soxR*; *CRP*; *emrB*; *ACC(6′)-Iy*; *baeR*; *bacA*; *msbA*; *E.coliUhpT*; *E.coliGlpT*
**Bull snake**	*Salmonella* 2	*S. enterica* subsp. *diarizonae*	Z:z35:z35	*marA*; *soxS*; *soxR*; *CRP*; *emrB*; *ACC(6′)-Iy*; *bacA*; *msbA*; *E.coliUhpT*; *E.coliGlpT*
**Small royal snake**	*Salmonella* 3	*S. enterica* subsp. *diarizonae*	51:k:z35	*marA*; *soxS*; *soxR*; *CRP*; *emrB*; *ACC(6′)-Iy*; *baeR*; *bacA*; *msbA*; *E.coliUhpT*; *E.coliGlpT*
**Gerrohsaurus** **major**	*Salmonella* 4	*S. enterica* subsp. *salamae*	F:g,m,s,t:1,5	*marA*; *soxS*; *soxR*; *CRP*; *emrB*; *arcB*; *mdtK*; *baeR*; *bacA*; *msbA*; *sdiA*; *E.coliUhpT*; *E.coliGlpT*
**False coral snake**	*Salmonella* 5	*S. enterica* subsp. *diarizonae*	Z:z35:z35	*marA*; *soxS*; *soxR*; *CRP*; *emrB*; *arcB*; *mdtK*; *ACC(6′)-Iy*; *baeR*; *bacA*; *msbA*; *sdiA*; *E.coliUhpT*; *E.coliGlpT*
**Coral snake**	*Salmonella* 6	*S. enteric* subsp. *diarizonae*	53:z10:z35	*marA*; *soxS*; *soxR*; *CRP*; *emrB*; *ACC(6′)-Iy*; *baeR*; *bacA*; *msbA*; *sdiA*; *E.coliUhpT*
**Pink boa**	*Salmonella* 7	*S. enterica* subsp. *enterica*	Muenchen	*marA*; *soxS*; *soxR*; *CRP*; *acrB*; *mdtK*; *ACC(6′)-Iy*; *baeR*; *bacA*; *msbA*; *sdiA*; *E.coliUhpT*; *E.coliGlpT*
**Royal python 4**	*Salmonella* 8	*S. enterica* subsp. *houtenae*	44:z4,z23:-	*marA*; *CRP*; *acrB*; *ACC(6′)-Iy*; *baeR*; *bacA*; *msbA*; *E.coliUhpT*
**Royal python 2**	*Salmonella* 9	ND	ND	*marA*; *soxS*; *soxR*; *CRP*; *emrB*; *ACC(6′)-Iy*; *baeR*; *bacA*; *msbA*; *E.coliUhpT*; *E.coliGlpT*

Serotype = sequence typing from public databases for molecular typing and microbial genome diversity (PubMLST), in accordance with the *Salmonella* nomenclature in use at CDC (Brenner et al., 2000); ND = No Identification. ^1^ Category of antibiotics and relative antibiotics resistance genes detected: β-lactam resistance genes = *marA*; *soxS*; *soxR*; *axrB*. Macrolide resistance gene = *CRP*. Carbapenem resistance genes = *soxS*; *marA*; CRP; *acrB*. Aminoglycoside resistance gene = *ACC(6′)-Iy*; *baeR*. Quinolone resistance gene = *soxS*; *soxR*; CRP; *emrB*; *marA*; *arcB*; *mdtK*. Rifampicin resistance gene = *soxS*; *soxR*; *marA*; *acrB*. Tetracycline resistance gene = *soxS*; *soxR*; *marA*; *acrB*. Aminocoumarin resistance genes = *baeR*. Nitroimidazole resistance genes = *msbA*. Fosfomycin resistance gene = *E. coliUhpT*; *E.coliGlpT*. Phenicol resistant gene = *soxS*; *soxR*; *marA*; *acrB*. Multidrug resistance gene = *mar*A. Multiefflux pump gene = *acrB*; *armB*; CRP; *mdtK*; *baeR*; *msbA*. Disinfectant agents and antiseptic resistance genes = *sdiA*; *soxS*; *sorR*; *marA*; *acrB*.

**Table 3:** Minimum inhibitory concentration (MIC; µL/mL) and minimum bactericidal concentration (MBC; µL/mL) of TEO upon nine strains of *S. enterica*, in the logarithmic phase, and the stationary phase. The values correspond to the MIC and MBC expressed as percentage values of TEO (*v*/*v*).

**Salmonella Strain**	**Logarithmic Phase 24 h**		**Logarithmic Phase 48 h**		**Logarithmic Phase Ratio**
	**MIC**	**MBC**	**MIC**	**MBC**	**MBC/MIC**
***Salmonella* 1**	0.312	0.312	0.078	0.312	1.6
***Salmonella* 2**	0.156	0.156	0.156	0.156	1
***Salmonella* 3**	0.156	0.156	0.156	0.156	1
***Salmonella* 4**	0.156	0.156	0.078	0.156	1
***Salmonella* 5**	0.156	0.156	0.078	0.156	1.3
***Salmonella* 6**	0.156	0.156	0.078	0.156	1.3
***Salmonella* 7**	0.156	0.312	0.156	0.312	2
***Salmonella* 8**	0.156	0.156	0.078	0.156	1.3
***Salmonella* 9**	0.078	0.078	0.078	0.078	1
***Salmonella Strain***	**Stationary Phase 24 h**		**Stationary Phase 48 h**		**Stationary Phase Ratio**
	**MIC**	**MBC**	**MIC**	**MBC**	**MBC/MIC**
***Salmonella* 1**	0.312	0.312	0.156	0.312	1.3
***Salmonella* 2**	0.156	0.156	0.156	0.156	1
***Salmonella* 3**	0.156	0.156	0.156	0.156	1
***Salmonella* 4**	0.156	0.156	0.078	0.156	1.3
***Salmonella* 5**	0.156	0.156	0.078	0.156	1.3
***Salmonella* 6**	0.156	0.156	0.078	0.156	1.3
***Salmonella* 7**	0.312	0.312	0.156	0.312	1.3
***Salmonella* 8**	0.156	0.156	0.078	0.156	1.3
***Salmonella* 9**	0.078	0.078	0.078	0.078	1

**Table 4:** Antibacterial activity of TEO against *Salmonella*, measured using agar diffusion testing, for the logarithmic phase, and the stationary phase.

**% TEO**	**Alone Inhibition Diameter (mm) (Logarithmic Phase)**								
	***S.* 1**	***S.* 2**	***S.* 3**	***S.* 4**	***S.* 5**	***S.* 6**	***S.* 7**	***S.* 8**	***S.* 9**
**5**	14	10	14	9	8	10	15	13.5	15
**2.5**	12	9	12	9	n.i.	9	8	10	9
**1.25**	10	8	10	8	n.i.	8	7	9	7
**0.625**	8	7	9	7	n.i.	n.i.	n.i.	n.i.	n.i.
**0.312**	8	n.i.	8	n.i.	n.i.	n.i.	n.i.	n.i.	n.i.
**0.156**	n.i.	n.i.	n.i.	n.i.	n.i.	n.i.	n.i.	n.i.	n.i.
**0.078**	n.i.	n.i.	n.i.	n.i.	n.i.	n.i.	n.i.	n.i.	n.i.
**0.039**	n.i.	n.i.	n.i.	n.i.	n.i.	n.i.	n.i.	n.i.	n.i.
**% TEO**	**Alone Inhibition Diameter (mm) (Stationary Phase)**								
	***S.* 1**	***S.* 2**	***S.* 3**	***S.* 4**	***S.* 5**	***S.* 6**	***S.* 7**	***S.* 8**	***S.* 9**
**5**	12	11.75	13.25	10	9.5	14	16.75	13.5	12
**2.5**	9	9.5	10	9	n.i.	8.5	9.25	9	8.25
**1.25**	8.5	7.5	9,5	n.i	n.i.	n.i.	7.25	n.i.	n.i.
**0.625**	7.5	7	n.i.	n.i.	n.i.	n.i.	n.i.	n.i.	n.i.
**0.312**	7	n.i.	n.i	n.i.	n.i.	n.i.	n.i.	n.i.	n.i.
**0.156**	n.i.	n.i.	n.i.	n.i.	n.i.	n.i.	n.i.	n.i.	n.i.
**0.078**	n.i.	n.i.	n.i.	n.i.	n.i.	n.i.	n.i.	n.i.	n.i.
**0.039**	n.i.	n.i.	n.i.	n.i.	n.i.	n.i.	n.i.	n.i.	n.i.

% TEO = Thymus Essential Oil percentage values (*v*/*v*). *S*.: *Salmonella* strain. The values correspond to the zone of inhibition including the diameter of the paper disk (6 mm) at different percentage values of TEO (*v*/*v*). Reference values = < 12 mm (weak activity zone); 12 mm ≥ inhibition zone < 20 mm (intermediate activity); inhibition zone ≥ 20 mm (strong activity); n.i. = no inhibition (Hamed et al. [45]).

## Data Availability

Data is contained within the article or supplementary material.

## Supplementary Materials

The following are available online at https://www.mdpi.com/article/10.3390/pathogens12060804/s1, Table S1: Gas chromatography with mass spectrometry (GC/MS) analysis for chemical composition evaluation of TEO, Table S2: Antibiotic resistance and MIC values of the Salmonella strains isolated from reptiles.
