# Supplementary material for: Antibacterial and Biofilm Production Inhibition Activity of Thymus vulgaris L. Essential Oil against Salmonella spp. Isolates from Reptiles
Source: Pathogens. 2023 Jun 5;12(6):804. doi: 10.3390/pathogens12060804 (PMC10304798; doi:10.3390/pathogens12060804)
Supplement: Supplementary file 1 [file pathogens-12-00804-s001.zip › pathogens-2359427-supplementary.pdf]

Supplementary files

Table S1. Gas chromatography with mass spectrometry (GC/MS) analysis for chemical composition evaluation of TEO.

N	Components	LRI	AI	*Thymus vulgaris*	
				**Area ± SEM**	**SI/MS**
1	Ethyl propanoate	714	714	0.10 ± 0.010	86
3	cyclene	920	919	0.13 ± 0.090	94
4	α-pinene [a]	930	931	1.81 ± 0.100	95
5	camphene [a]	952	952	1.89 ± 0.110	96
6	β-thujene	968	968	0.71 ± 0.060	93
7	mushroom alcohol	975	975	0.37 ± 0.020	83
8	β-pinene[a]	982	980	0.56 ± 0.030	94
9	β-myrcene	990	991	1.42 ± 0.080	86
10	α-phellandrene[a]	1002	1003	0.15 ± 0.010	91
11	o-cymene	1021	1021	19.64 ± 1.500	95
12	eucalyptol [a]	1023	1023	0.89 ± 0.050	99
13	limonene[a]	1030	1032	0.60 ± 0.040	91
14	γ-terpinene[a]	1062	1064	8.83 ± 1.030	94
15	terpinolene	1083	1085	1.33 ± 0.880	97
16	β-linalool[a]	1100	1101	4.07 ± 1.020	97
17	camphor	1145	1146	1.69 ± 0.850	98
18	terpineol, cis-β-	1147	1147	0.13 ± 0.010	90
19	endo-borneol	1166	1167	1.85 ± 0.700	97
20	terpinen-4-ol	1171	1171	1.83 ± 0.830	96
21	α-terpineol	1178	1179	0.12 ± 0.010	86
22	anisole	1234	1235	0.39 ± 0.060	90
23	anisole, 2-isopropyl-4-methyl-	1244	1244	0.42 ± 0.050	94
24	thymol[a]	1290	1290	47.01 ± 3.320	94
25	caryophyllene[a]	1415	1415	2.18 ± 0.870	99
26	caryophyllene oxyde	1596	1592	0.58 ± 0.030	91
	% Characterized	/	/	98.70	/
	Others	/	/	1.30	/

Legend. [a]: standard compounds. Linear retention index (LRI) on HP-5MS column was experimentally determined using a homologous series of C10-C40 alkanes standard mixture (Van den Dool and Kratz, 1993). Arithmetic index (AI) was taken from Adams (2007) and/or the NIST 2017 Database (NIST 17, 2017. Mass Spectral Library (NIST/EPA/NIH). Relative percentage values are means of three determinations with a structural equation modeling (SEM) in all cases below 10%.

Table S2. Antibiotic resistance and MIC values of the *Salmonella* strains isolated from reptiles (*Salmonella* 1–9).

Antibiotic	Breakpoints			S1		S2		S3		S4		S5		S6		S7		S8		S9	
	S	I	R	MIC^	S/R	MIC^	S/R	MIC^	S/R	MIC^	S/R	MIC^	S/R	MIC^	S/R	MIC^	S/R	MIC^	S/R	MIC^	S/R
Amikacin - AMI	16	32	64	< 8	S	< 8	S	< 8	S	< 8	S	< 8	S	< 8	S	< 8	S	< 8	S	< 8	S
Piperacillin/Tazobactam	16/4	32/4 – 64/4	128/4	< 8/4	S	< 8/4	S	< 8/4	S	< 8/4	S	< 8/4	S	< 8/4	S	< 8/4	S	< 8/4	S	< 8/4	S
Ticarcillin/Clavulanic acid constant 2-TIM2	16/2	32/2 – 64/2	128/2	< 8/2	S	< 8/2	S	< 8/2	S	< 8/2	S	< 8/2	S	< 8/2	S	< 8/2	S	< 8/2	S	< 8/2	S
Levofloxacin - LEVO	0.5	1	2	< 1	S	< 1	S	< 1	S	< 1	S	< 1	S	< 1	S	< 1	S	< 1	S	< 1	S
Nitrofurantoin - NIT	32	64	128	< 32	S	< 32	S	< 32	S	< 32	S	< 32	S	< 32	S	< 32	S	< 32	S	< 32	S
Tetracycline - TET	4	8	16	< 4	S	< 4	S	< 4	S	< 4	S	< 4	S	< 4	S	< 4	S	< 4	S	< 4	S
Doripenem - DOR	1	2	4	< 0.5	S	< 0.5	S	< 0.5	S	< 0.5	S	< 0.5	S	< 0.5	S	< 0.5	S	< 0.5	S	< 0.5	S
Minocycline - MIN	4	8	16	2	S	2	S	2	S	2	S	2	S	4	S	4	S	4	S	2	S
Ertapenem - ETP	0.5	1	2	< 0.25	S	< 0.25	S	< 0.25	S	< 0.25	S	< 0,25	S	< 0.25	S	< 0.25	S	< 0,25	S	< 0.25	S
Trimethoprim /Sulfamethoxazole -SXT	2/38	-	4/76	< 2/30	S	< 2/30	S	< 2/30	S	< 2/30	S	< 2/30	S	< 2/30	S	< 2/30	S	< 2/30	S	< 2/30	S
Imipenem - IMI	1	2	4	< 0.5	S	< 0.5	S	< 0.5	S	< 0.5	S	< 0.5	S	< 0.5	S	< 0.5	S	< 0.5	S	< 0.5	S
Piperacillin - PIP	16	32–64	128	< 16	S	< 16	S	< 16	S	< 16	S	< 16	S	< 16	S	< 16	S	< 16	S	< 16	S
Meropenem - MERO	1	2	4	< 0.5	S	< 0.5	S	< 0.5	S	< 0.5	S	< 0.5	S	< 0.5	S	< 0.5	S	< 0.5	S	< 0.5	S
Gentamicin - GEN	4	8	16	< 2	S	< 2	S	< 2	S	< 2	S	< 2	S	< 2	S	< 2	S	< 2	S	< 2	S
Cefazolin - FAZ	2	4	8	2	S	2	S	2	S	2	S	2	S	2	S	2	S	5	s	2	S
Tobramycin - TOB	4	8	16	< 2	S	< 2	S	< 2	S	< 2	S	< 2	S	< 2	S	< 2	S	< 2	S	< 2	S
Ceftazidime - TAZ	4	8	16	< 1	S	< 1	S	< 1	S	< 1	S	< 1	S	< 1	S	< 1	S	< 1	S	< 1	S
Ampicillin / Sulbactam	8/4	16/8	32/16	< 4/2	S	< 4/2	S	< 4/2	S	< 4/2	S	< 4/2	S	< 4/2	S	< 4/2	S	< 4/2	S	< 4/2	S
Aztreonam - AZT	4	8	16	< 1	S	< 1	S	< 1	S	< 1	S	< 1	S	< 1	S	< 1	S	< 1	S	< 1	S
Ampicillin - AMP	8	16	32	< 8	S	< 8	S	< 8	S	< 8	S	< 8	S	< 8	S	< 8	S	< 8	S	< 8	S
Cefepime - FEP	2	4–8	16	< 4	S	< 4	S	< 4	S	< 4	S	< 4	S	< 4	S	< 4	S	< 4	S	< 4	S
Ciprofloxacin - CIP	0.25	0.5	1	< 0.5	S	< 0.5	S	< 05	S	< 0.5	S	< 0.5	S	< 0.5	S	< 0.5	S	< 0.5	S	< 0.5	S
Ceftriaxone - AXO	1	2	4	< 0.5	S	< 0.5	S	< 0.5	S	< 0.5	S	< 0.5	S	< 0.5	S	< 0.5	S	< 0.5	S	< 0.5	S

^µg/mL; S: Susceptible; I: Intermediate; R: Resistant.
